# Impact of *DCC* (rs714) and *PSCA* (rs2294008 and rs2976392) Gene Polymorphism in Modulating Cancer Risk in Asian Population

**DOI:** 10.3390/genes7020009

**Published:** 2016-02-16

**Authors:** Vishal Chandra, Jong Joo Kim, Usha Gupta, Balraj Mittal, Rajani Rai

**Affiliations:** 1 Department of Biosciences, Integral University, Lucknow 226026 (Uttar Pradesh), India; chandra_vishal@rediffmail.com; 2 School of Biotechnology, Yeungnam University, Gyeongsan, Gyeongbuk 712-749, Korea; kimjj@ynu.ac.kr; 3 Department of Genetics, Sanjay Gandhi Post Graduate Institute of Medical Sciences (SGPGIMS), Lucknow 226014 (Uttar Pradesh), India; guptaushaa@gmail.com(U.G.); balraj@sgpgi.ac.in (B.M.)

**Keywords:** *PSCA*, *DCC*, polymorphism, cancer, meta-analysis

## Abstract

Multiple studies have investigated the association of gene variant of *Deleted in colorectal carcinoma (DCC)* and *Prostate Stem cell antigen (PSCA)* with various cancer susceptibility; however, the results are discrepant. Since SNPs are emerging as promising biomarker of cancer susceptibility, here, we aimed to execute a meta-analysis of *DCC* (rs714 *A* > *G*) and *PSCA* (rs2294008 *C* > *T*, rs2976392 *G* > *A*) polymorphism to demonstrate the more accurate strength of these associations. We followed a rigorous inclusion/exclusion criteria and calculated the pooled odds ratios (ORs) and 95% confidence intervals (CIs). Overall, the pooled analysis showed that the *DCC* rs714 conferred increased risk of cancer only in Asians (*AA vs.* *GG*: OR = 1.86, *p* ≤ 0.0001; *AG vs.* *GG*: OR = 1.43, *p* = 0.005; *GA* + *AA vs.* *GG*: OR = 1.66, *p* ≤ 0.0001; *AA vs.* *GG* + *GA*; OR = 1.52, *p* ≤ 0.004, *A vs.* *G* allele: OR = 1.41, *p* ≤ 0.0001). *PSCA* rs2294008 was associated with increased overall cancer risk (*TT vs.* *CC*: OR = 1.28, *p* = 0.002; *CT vs.* *CC*: OR = 1.21, *p* ≤ 0.0001; *CT* + *TT vs.* *CC*: OR = 1.24, *p* ≤ 0.0001; *TT vs.* *CC* + *CT*; OR = 1.17, *p* ≤ 0.005, *T vs.* *C* allele: OR = 1.16, *p* ≤ 0.0001); however, in stratified analysis this association was limited only to gastric and bladder cancer and the strength was more prominent in Asians. In contrast, the *PSCA* rs2976392 SNP did not modulate the cancer risk. Therefore, we concluded that rs714 and rs2294008 polymorphism may represent a potential genetic biomarker for cancer risk in Asians and gastric as well as bladder cancer, respectively. However, since our study is limited to Asians and cancer types, further larger studies involving other cancers and/or population, gene-environment interactions and the mechanism of *DCC* and *PSCA* gene deregulation are desired to define the role of genotype with overall cancer risk.

## 1. Introduction

Cancer initiation and progression is a complex and multifaceted process involving numerous genetic as well as environmental risk factors [1]. Moreover, inheritance of the majority of cancers is polygenic, and several genes with mild consequence are involved in the carcinogenesis [2]. Multiple studies (Genome wide association studies/GWAS, case-control and cohort) have unveiled single-nucleotide polymorphisms (SNPs) as the most common forms of human genetic variation that may affect individual’s susceptibility to cancer. Further, emerging evidence has shown that SNPs may be used as promising biomarker of individual genetic background to envisage therapeutic responses and prognosis in cancer patients, thus representing an interesting field of cancer research [3,4].

The *deleted in colorectal carcinoma* (*DCC*) is a well familiar tumor suppressor gene that functions in cell migration, cell cycle arrest and apoptosis, and has been found to be frequently deregulated or inactivated in various cancers [5,6,7]. Loss of heterozygosity (LOH), the most common genetic alteration of the DCC gene, is established to be implicated in pathogenesis of various cancers [8,9]. Further, *DCC* gene variants have been associated with increased susceptibility of various cancers. *DCC* rs714 *A* > *G* polymorphism, the most widely studied SNP of *DCC* gene, is LOH marker associated with decreased expression of DCC and with increased risk of colorectal and gallbladder cancer [10,11,12,13]. However, the published articles have generally been confined in terms of sample size, ethnicity and study designs.

Prostate stem cell antigen (PSCA) is a member of Ly-6/Thy-1 family of glycosylphosphatidyl-inositol (GPI)-anchored cell-surface proteins having a crucial role in cell adhesion, proliferation, and survival [14]. PSCA was found to be aberrantly expressed in several human cancers, and since it has restricted expression in normal tissues, PSCA represents an ideal target for cancer diagnosis and therapy [15,16,17,18,19,20,21]. Human *PSCA* gene maps on chromosome *8q24.2* containing 464 SNPs. rs2294008 *C* > *T* and rs2976392 *G* > *A* are the most extensively studied SNPs in the *PSCA* gene shown to be associated with increased risk of bladder and stomach cancer [22,23]. However, in our previous study, we failed to find an association between rs2294008 SNP and gallbladder cancer risk [24]. A number of studies have also investigated the association of these SNPs with various cancer susceptibility, though the results are discrepant as the *PSCA* gene function in a tissue/organ specific manner, *i.e.*, act as an oncogene in some cancers while tumor suppressor gene in others [25,26,27,28].

Considering the panoptic role of *DCC* (rs714) and *PSCA* (rs2294008, rs2976392) polymorphism in the carcinogenesis, and increasing number of reports on different cancer in recent years, there is a prerequisite to reconcile all the discordant results to clarify its role in cancer susceptibility. Since meta-analysis represents an effective way to merge information from several studies dealing with the same concern, we performed a meta-analysis of all eligible case-control studies to better interpret the associations between these SNPs and cancer.

## 2. Materials and Methods

We adopted the statement of PRISMA for reporting meta-analysis [29].

### 2.1. Literature Search

A systematic and comprehensive literature search was performed from electronic database to find all the published case-control studies on the association of *DCC* (rs714 *A* > *G*) and *PSCA* (rs2294008 *C* > *T* and rs2976392 *G* > *A*) polymorphism with cancer susceptibility until September 2015. The search strategies were without time or geographical restriction, but limited to human-associated studies and English language papers. The “Pubmed”, “Medline”, “Google Scholar”, “EMBASE”, and “Scopus” database were examined using the following MeSH index keywords: “prostate stem cell antigen”, “*DCC* rs714 (*A* > *G*)”, “*PSCA* rs2294008 (*C* > *T*) and/or rs2976392 (*G* > *A*)”, “single nucleotide polymorphism (SNP)/variation/genotype”, in combination with “Cancer/carcinoma” or “tumor”. The titles and abstracts of potential articles were sorted to achieve their relevancy, and irrelevant studies were left off. Additional relevant studies were collected through manual examination of reference list of the retrieved articles and previous reviews on the topic.

### 2.2. Study Selection

The selection criteria of the study were (1) original case-control study accounting the association of *DCC* (rs714 *A* > *G*) and *PSCA* (rs2294008 *C* > *T* or rs2976392 *G* > *A*) polymorphism with cancer; (2) studies with sufficient information to estimate the relative risk and 95% confidence intervals (CI); (3) enlisting pathologically confirmed incident cancer cases; (4) studies including only cancer-free (healthy) controls; (5) concordance of genotypic frequencies with Hardy-Weinberg equilibrium (HWE) in controls.

The major exclusion criteria were as follows: (1) ecological studies, case reports, reviews, abstract, comment and editorials; (2) articles published in a language other than English; (3) lack of control population; (4) studies with benign, hyperplasia or other related pre-malignant taken as controls; (5) insufficient data; (6) duplicate studies; (7) not for cancer research; (8) not in accordance with Hardy-Weinberg equilibrium in control groups.

### 2.3. Data Extraction

The qualification evaluation of each eligible study was carried out by two investigators separately and the information was cautiously extracted from all eligible publications according to the inclusion and exclusion criteria listed above. Any disagreements were further discussed and resolved by consensus.

Data including first author name, year of publication, country of origin, ethnicity, genotyping methods, cancer types, frequency of cases and controls, genotype frequency, minor allelic frequencies, *etc.,* were extracted from each study. If identical data were reported in more than one publication or had previously been reported somewhere else, only the original report with the largest sample size was included. Articles covering different ethnic groups and different countries or different cancer were viewed as different studies for each category cited above. Subgroup analysis, stratified by cancer type and ethnicity was also performed. Those cancer types appeared in only one or two studies, were placed into the “other cancers” subgroup. Ethnicity was classified as Caucasian, Asian and Mixed.

### 2.4. Statistical Analysis

The intensity level of association between studied SNPs and cancer susceptibility was assessed by computing crude ORs and corresponding 95% CI. The pooled ORs was estimated for allele contrast, log-additive, dominant, and recessive models. Deviation from HWE was analyzed by using the Chi-square goodness of fit test (significant at the 0.05 level). The analyses were stratified on the basis of cancer types (gastric cancer/GC, bladder cancer/BC and others) and ethnicity (Asian and Caucasian). Chi-square-based Q statistics was calculated to evaluate the heterogeneity across the studies, and it was considered significant at *p* < 0.05. Heterogeneity was measured using the *I*^2^ value, the percentage of variation across studies that are due to heterogeneity rather than chance. The value of *I*^2^ = 0%, 25%, 50% and 75% represent no observed heterogeneity, low, moderate, and high heterogeneity, respectively [30]. The pooled ORs were calculated by the fixed-effect model in case of no heterogeneity [31] otherwise, a random-effect model was used [32]. Moreover, a sensitivity analysis was performed to check if the alteration of inclusion criteria affects the results of the meta-analysis. For this, the meta-analysis estimates were computed after excluding one study at a time. The publication bias was assessed graphically using Funnel plot, and the plot asymmetry was investigated by Egger test [33] and *p* < 0.05 was considered as statistically significant publication bias. All of the statistical analyses were done by Comprehensive Meta-analysis software (Version 2.0, BIOSTAT, Englewood, NJ, USA).

## 3. Result

### 3.1. Study Characteristics

According to the search strategy mentioned above, only four articles were found eligible for *DCC* (rs714 *A* > *G*) meta-analysis [10,12,13,34]. Among them, Malik *et al.* (2013) analyzed the association of rs714 polymorphism with gastric cancer (GC) and esophageal cancer (EC), and hence these were counted as two different studies [12]. Thus a total of five studies from four articles with a total of 1018 multiple cancer cases and 952 controls was included for *DCC* rs714 *A* > *G* meta-analysis.

**Figure 1:**
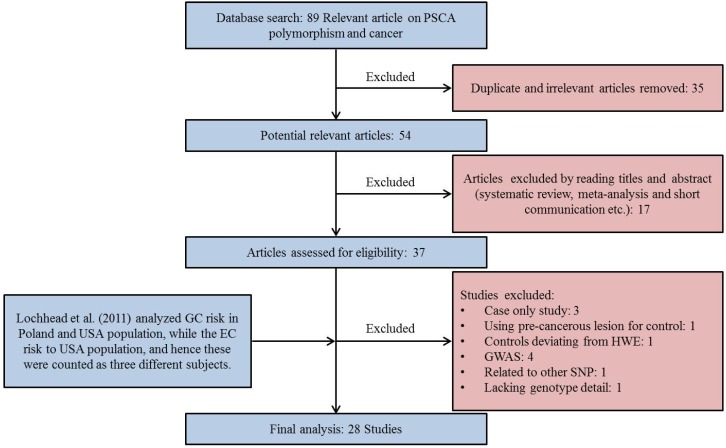
Flow chart of study selection for *Prostate Stem cell antigen (PSCA)* rs2294008 polymorphism. The study by Lochhead *et al.* (2011) [42] involved three case-control studies out of which one study was excluded because of deviation from Hardy-Weinberg equilibrium (HWE) in control population, so the number of case-control studies are different than number of articles included in the meta-analysis.

For *PSCA* meta-analysis, a total of 27 articles ([24,28,35,36,37,38,39,40,41,42,43,44,45,46,47,48,49,50,51,52,53,54,55,56,57,58] Figure 1) were found eligible. Remarkably, the study by Lochhead *et al.* (2011) analyzed the association of rs2294008 polymorphism with GC risk in Poland and USA population, while the EC risk to USA population, and hence these were counted as three different studies [42]. However, the frequency of *PSCA* SNP deviated from HWE in Poland control subjects, hence excluded. Finally, 28 studies from 27 articles met our established inclusion criteria for rs2294008 with a total of 17,479 multiple cancer cases and 19,799 controls. Among them only 11 study (with a total of 5970 multiple cancer cases and 5707 controls) investigated the rs2976392 polymorphism in various cancers [27,36,37,38,41,44,47,53,54,57,58].

The characteristics of eligible studies included in the analysis are presented in Table 1. All studies were retrospective case-control studies using validated genotyping methods and genotype frequencies in the control cohort were in accordance with Hardy-Weinberg equilibrium (HWE).

### 3.2. Quantitative Synthesis

**Figure 2:**
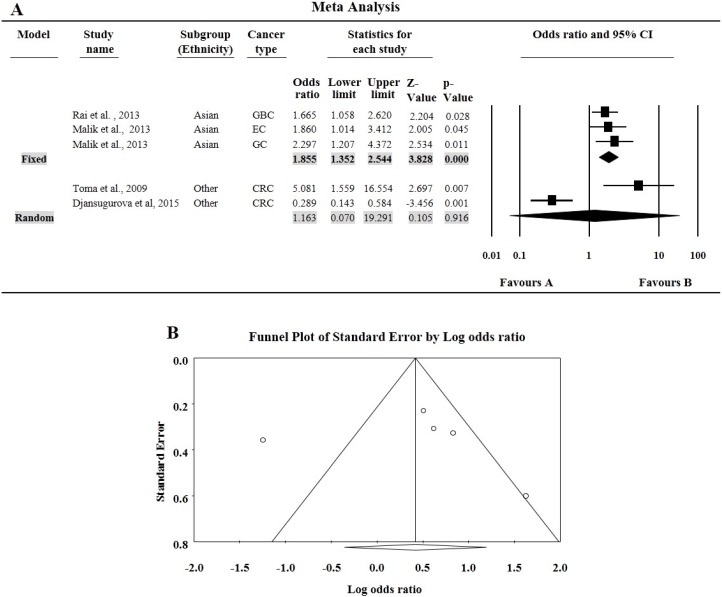
(**A**) Forest plots for meta-analysis of *DCC* rs714 polymorphism (*AA vs. GG*) and cancer risk after ethnicity based stratification. For each study, the estimates of odds ratio (OR) and 95% confidence interval (CI) were plotted with square and horizontal line. The size of the square points is the relative weight of the respective study. Diamond indicates the pooled OR and its 95% CI; (**B**) Funnel plot analysis to detect publication bias for the Delet*ed in colorectal carcinoma* (*DCC)* rs714 polymorphism (*AA vs.* *GG*) and overall cancer risk. Each dot represents an individual study for the indicated association. Areas of squares of individual studies are inversely proportional to the variance of the log odds ratios and the horizontal lines represent CIs.

The minor allele frequency (MAF) for rs714 SNP varies from 28% to 38% (Table 2). For rs714 *A* > *G* polymorphism, none of the genotypic combination was found to affect the risk of overall cancer compared with the wild genotype. Since there are only five studies we did not perform subgroup analysis except for ethnicity. In Asian subgroups, having three studies with a total number of 649 multiple cancer cases and 650 controls, all the four genotypic model were found to significantly associated with increased risk of cancer (*A vs.* *G*: OR = 1.41, 95% CI = 1.20–1.66, *p* ≤ 0.000; *AA vs.* *GG*: OR = 1.86, 95% CI = 1.35–2.54, *p* ≤ 0.000; *GA vs.* *GG*: OR = 1.43, 95% CI = 1.11–1.85, *p* = 0.005; *GA* + *AA vs.* *GG*: OR = 1.66, 95% CI = 1.31–2.09, *p* ≤ 0.000; *AA vs.* *GG* + *GA*: OR = 1.52, 95% CI = 1.14–2.03, *p* = 0.004, Figure 2A.

**Table 1:** Studies included in meta-analysis.

SN	Author	Reference	Country	Ethnicity	Cancer Type	Case/Control	*p*_HWE_	MAF	Genotyping Method
***DCC***** rs714 (*A* > *G*)**									
**1**	Toma *et al.*, 2009	[34]	Romania	Caucasian	CRC	120/60	0.603	0.28	PCR-RFLP
**2**	Rai *et al.*, 2013	[10]	India	Asian	GBC	406/260	0.062	0.38	PCR-RFLP
**3**	Malik *et al.*, 2013	[12]	India	Asian	EC	135/195	0.187	0.36	PCR-RFLP
**4**	Malik *et al.*, 2013	[12]	India	Asian	GC	108/195	0.187	0.36	PCR-RFLP
**5**	Djansugurova *et al.*, 2015	[13]	Kazakhstan	Mixed	CRC	249/242	0.187	0.36	PCR-RFLP
***PSCA***** rs2294008 (*C* > *T*)**									
**1**	Wu *et al.*, 2009	[36]	China	Asian	GC	1736/1020	0.587	0.28	PCR-RFLP
**2**	Matsuo *et al.*, 2009	[27]	Japan	Asian	GC	708/708	0.638	0.38	Taqman
**3**	Wang *et al.*, 2010	[28]	China	Asian	BC	581/580	0.508	0.27	PCR-RFLP
**4**	Lu *et al.*, 2010	[37]	China	Asian	GC	1053/1100	0.166	0.25	PCR-RFLP
**5**	Ou *et al.*, 2010	[38]	China	Asian	GC	196/246	0.924	0.27	PCR-LDR
**6**	Zeng *et al.*, 2011	[39]	China	Asian	GC	460/549	0.493	0.27	PCR-RFLP
**7**	Song *et al.*, 2011	[40]	Korea	Asian	GC	3245/1700	0.131	0.48	PCR-RFLP
**8**	Joung *et al.*, 2011	[41]	Korea	Asian	PC	194/169	0.963	0.47	MASS ARRAY
**9**	Lochhead *et al.*, 2011	[42]	USA	Caucasian	EC	159/211	0.405	0.5	Taqman
**10**	Lochhead *et al.*, 2011	[42]	USA	Caucasian	GC	309/211	0.405	0.5	Taqman
**11**	Sala *et al.*, 2012	[43]	Europe	Caucasian	GC	411/1530	0.088	0.44	SNP ARRAY
**12**	Kim *et al.*, 2012	[44]	Korea	Asian	BrC	456/461	0.324	0.49	MALDI-TOF MS
**13**	Smith *et al.*, 2012	[45]	Scotland	Caucasian	CRC	77/804	0.981	0.4	Taqman
**14**	Li *et al.*, 2012	[46]	China	Asian	GC	300/300	0.65	0.26	MASS-ARRAY IPLEX
**15**	Ono *et al.*, 2013	[47]	Japan	Asian	GBC	44/173	0.242	0.39	Taqman
**16**	Ma *et al.*, 2013	[48]	China	Asian	BC	184/962	0.562	0.25	MASS-ARRAY IPLEX
**17**	Zhao *et al.*, 2013	[35]	China	Asian	GC	717/951	0.913	0.3	PCR-DHPLC
**18**	Rai *et al.*, 2013	[24]	India	Asian	GBC	405/247	0.492	0.43	Taqman
**19**	Dai *et al.*, 2014	[49]	China	Asian	EC	2083/2220	0.944	0.27	Taqman
**20**	Sun *et al.*, 2014	[50]	Texas	Caucasian	GC	132/125	0.926	0.49	Taqman
**21**	Wang *et al.*, 2014	[51]	China	Asian	BC	1210/1008	0.739	0.25	Taqman
**22**	Lee *et al.*, 2014	[52]	Korea	Asian	BC	411/1700	0.13	0.48	HRM
**23**	Kupcinskas *et al.*, 2014	[53]	Lithuania	Caucasian	GC	252/246	0.834	0.48	Taqman
**24**	Sun *et al.*, 2015	[54]	China	Asian	GC	692/774	0.105	0.28	Taqman
**25**	MA *et al.*, 2015	[55]	Spain	Caucasian	GC	603/675	0.349	0.45	Taqman
**26**	Ichikawa *et al.*, 2015	[56]	Japan	Asian	GC	193/266	0.185	0.42	PCR-RFLP
**27**	Zhang *et al.*, 2015	[57]	China	Asian	GC	476/481	0.617	0.27	MASS ARRAY
**28**	Kupcinskas *et al.*, 2015	[58]	Latvia	Caucasian	CRC	192/382	0.943	0.48	Taqman
	***PSCA***** rs2976392 (*G* > *A*)**								
**1**	Wu *et al.*, 2009	[36]	China	Asian	GC	1724/1002	0.35	0.29	PCR-RFLP
**2**	Matsuo *et al.*, 2009	[27]	Japan	Asian	GC	707/707	0.635	0.37	Taqman
**3**	Lu *et al.*, 2010	[37]	China	Asian	GC	1043/1082	0.336	0.26	PCR-RFLP
**4**	Ou *et al.*, 2010	[38]	China	Asian	GC	196/246	0.298	0.26	PCR-LDR
**5**	Joung *et al.*, 2011	[41]	Korea	Asian	PC	194/168	0.848	0.47	MASS ARRAY
**6**	Kim *et al.*, 2012	[44]	Korea	Asian	BrC	453/460	0.397	0.49	MALDI-TOF MS
**7**	Ono *et al.*, 2013	[47]	Japan	Asian	GBC	44/173	0.328	0.61	Taqman
**8**	Kupcinskas *et al.*, 2014	[53]	Lithuania	Caucasian	GC	249/232	0.986	0.48	Taqman
**9**	Sun *et al.*, 2015	[54]	China	Asian	GC	692/774	0.13	0.29	Taqman
**10**	Zhang *et al.*, 2015	[57]	China	Asian	GC	476/481	0.939	0.28	MASS ARRAY
**11**	Kupcinskas *et al.*, 2015	[58]	Latvia	Caucasian	CRC	192/382	0.856	0.48	Taqman

GC—Gastric cancer, BC—Bladder cancer, GBC—Gallbladder cancer, EC—Esophageal cancer, BrC—Breast Cancer, CRC—Colorectal cancer, PC—Prostate Cancer, HWE Hardy—Weinberg equilibrium, MAF—Minor allelic frequency, PCR—Polymerase chain reaction, RFLP—Restriction fragment length polymorphism, LDR—Ligation detection reaction, DHPLC—Denaturing high performance liquid chromatography, HRM—High-resolution melting, SNP—Single nucleotide polymorphism, MALDI-TOF-MS—Matrix Assisted Laser Desorption/Ionization Time of Flight Mass Spectrometry.

**Figure 3:**
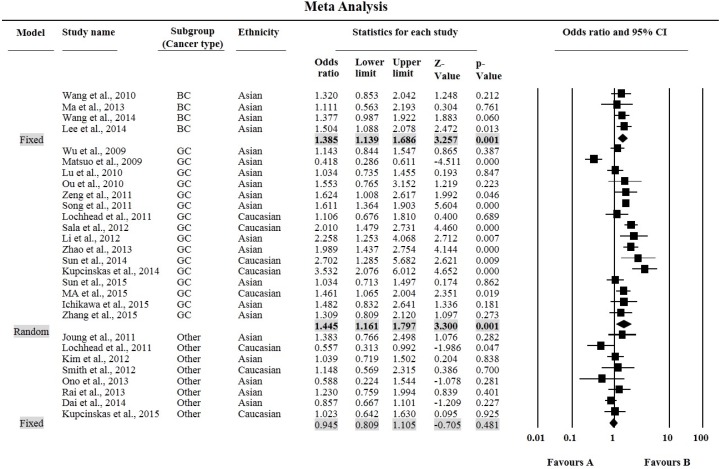
Forest plots for meta-analysis of *PSCA* rs2294008 *C* > *T* polymorphism (*TT vs. CC*) and cancer risk after cancer site based stratification. For each study, the estimates of OR and 95% CI were plotted with square and horizontal line. The size of the square points is the relative weight of the respective study. Diamond indicates the pooled OR and its 95% CI.

The MAF for *PSCA* rs2294008 polymorphism varies as 25%–49% in Asians, and 40%–50% in Caucasians. The results of our meta-analysis are shown in Table 3. Overall, the individuals carrying the *TT* or *CT* genotype were at an increased risk of cancer compared with the *CC* genotype (*TT*: OR = 1.28, 95% CI = 1.10–1.50, *p* = 0.002, Figure 3 and *CT*: OR = 1.21, 95% CI = 1.09–1.34, *p* ≤ 0.0001, respectively, Table 3.). Moreover, significant associations were also found in *T vs.* *C* allele (OR = 1.16, 95% CI = 1.07–1.25, *p* ≤ 0.0001.), as well as in dominant (*CT* + *TT vs.* *CC*: OR = 1.24, 95% CI = 1.11–1.39, *p* ≤ 0.0001) and recessive models (*TT vs.* *CC* + *CT*: OR = 1.17, 95% CI = 1.05–1.30, *p* = 0.005), in the pooled analyses. When stratifying by cancer type, significantly increased risk was limited to gastric cancer and bladder cancer in all genetic models except for the recessive model for bladder cancer. Further, subgroup analyses based on ethnicity showed that rs2294008 polymorphism modulate the risk of cancer in Caucasian ethnicity with only *TT* genotype and recessive model, while in the Asian ethnicity subgroup, all genetic models (except recessive model) were associated with increased cancer risk (Table 3).

**Table 2:** Meta-analysis Result for *DCC* rs714 *A* > *G* polymorphism.

Variables	*N*	Case/Control	*A vs.* *G* Allele			*AA vs.* *GG*			*GA vs.* *GG*			*GA* + *AA vs.* *GG*			*AA vs.* *GG* + *GA*		
			OR (95% CI)	*p*	*p*_h_/*I*^2^	OR (95% CI)	*p*	*p*_h_/*I*^2^	OR (95% CI)	*p*	*p*_h_/*I*^2^	OR (95% CI)	*p*	*p*_h_/*I*^2^	OR (95% CI)	*p*	*p*_h_/*I*^2^
**All**	5	1018/952	1.31 (0.93–1.86)	0.121	0.000/84.033	1.52 (0.70–3.3)	0.289	0.000/85.434	1.37 (0.98–1.92)	0.068	0.032 62.210	1.49 (0.98–2.28)	0.063	0.001/79.104	1.27 (0.64–2.52)	0.495	0.000/84.117
**Ethnicity**																	
**Caucasian**	1	120/60	2.14 (1.34–3.43)	**0.002**	1.000/0.000	5.08 (1.56–16.55)	**0.007**	1.000/0.000	2.53 (1.29–4.97)	**0.007**	1.000/0.000	2.87 (1.50–5.50)	**0.001**	1.000/0.000	2.97 (0.97–9.09)	0.056	1.000/0.000
**Asian**	3	649/650	1.41 (1.20–1.66)	**0.000**	0.810/0.000	1.86 (1.35–2.54)	**0.000**	0.725/0.000	1.43 (1.11–1.85)	**0.005**	0.107 55.174	1.66 (1.31–2.09)	**0.000**	0.182/41.288	1.52 (1.14–2.03)	**0.004**	0.193/39.43
**Mixed**	1	249/242	0.70 (0.54–0.92)	**0.011**	1.000/0.000	0.29 (0.14–0.58)	**0.001**	1.000/0.000	0.99 (0.68–1.44)	0.975	1.000/0.000	0.81 (0.57–1.16)	0.246	1.000/0.000	0.29 (0.15–0.57)	**0.000**	1.000/0.000

Significant associations are shown in bold, ***p***h—*p* value of Q test for heterogeneity, OR—Odds Ratio, CI—Confidence Interval.

**Figure 4:**
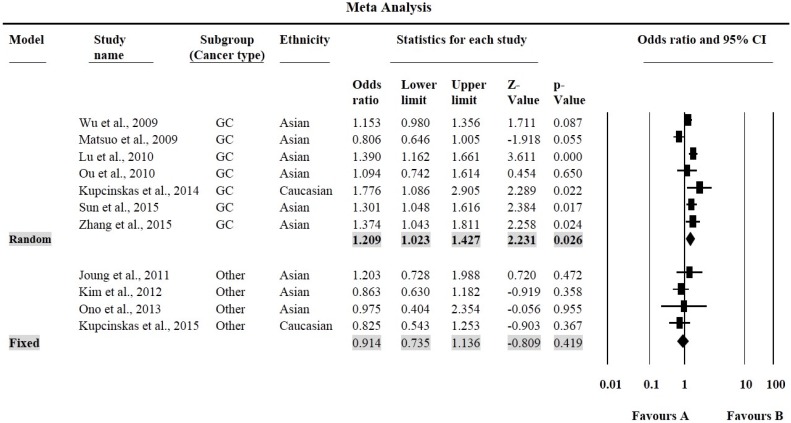
Forest plots for meta-analysis of *PSCA* rs2976392 *G* > *A* polymorphism (*GA vs.* *GG*) and cancer risk stratified by cancer site. For each study, the estimates of OR and 95% CI were plotted with square and horizontal line. The size of the square points is the relative weight of the respective study. Diamond indicates the pooled OR and its 95% CI.

The MAF for rs2976392 SNP varies from 26% to 48% (Table 4). For, rs2976392 *G* > *A* polymorphism, individuals carrying the *GA* genotype were at an increased risk of only GC cancer (OR = 1.21, 95% CI = 1.02–1.43, *p* = 0.026, Figure 4).

### 3.3. Tests of Heterogeneity and Sensitivity Analysis

The present meta-analysis revealed significant heterogeneity for all studied SNPs. For rs714 polymorphism, the removal of the study by Djansugurova *et al.* (2015) [13] was found to remove heterogeneity in all genotype models (*AA vs.* *GG*: *p*h = 0.354, *I*^2^ = 7.759; *GA vs.* *GG*: *p*h = 0.077, *I*^2^ = 56.193; *GA* + *AA vs.* *GG*: *p*h = 0.119, *I*^2^ = 48.666; *AA vs.* *GG* + *GA*: *p*h = 0.205, *I*^2^ = 34.523; *A vs.* *G*: *p*h = 0.376, *I*^2^ = 3.346). However, it was found to significantly change the pooled result.

For *PSCA* polymorphism, our sensitivity analysis showed that removal of the studies by Lochhead *et al.* (2011) [42], Matsuo *et al.* (2009) [27], Kupcinskas *et al.* (2014) [53] and Dai *et al.* (2014) [49] collectively abolished heterogeneity at the allele (*T vs.* *C*: ***p***h = 0.056, *I*^2^ = 34.210) and heterogenotype level (*CT vs.* *CC*: *p*h = 0.090, *I*^2^ = 29.731), without significantly influencing the pooled ORs of the overall cancer risk. Similarly, Dai *et al.* (2014) [49], Kupcinskas *et al.* (2014) [53], Kupcinskas *et al.* (2015) [58], Lochhead *et al.* (2011) [42] and Matsuo *et al.* (2009) [27] seemed to be responsible for heterogeneity at variant level (*TT vs. CC*: *p*h = 0.053, *I*^2^ = 35.265) as well as in dominant model (*CT* + *TT vs.* *CC*: *p*h = 0.098, *I*^2^ = 29.281) while only Kupcinskas *et al.* (2014) [53] and Matsuo *et al.* (2009) [27] were responsible for heterogeneity in recessive models (*TT vs.* *CC* + *CT*: ***p***h = 0.143, *I*^2^ = 25.154). Further, our sensitivity analysis affirmed the consistency of the results and the corresponding pooled ORs were not significantly altered by any single study in the entire four genetic models .

**Table 3:** Meta-analysis Result for *PSCA* rs2294008 *C* > *T* polymorphism.

Variables	*N*	Case/Control	*T vs.* *C* Allele			*TT vs.* *CC*			*CT vs.* *CC*			*CT* + *TT vs.* *CC*			*TT vs.* *CC* + *CT*		
			OR (95% CI)	*p*	*p*_h_/*I*^2^	OR (95% CI)	*p*	*p*_h_/*I*^2^	OR (95% CI)	*p*	*p*_h_/*I*^2^	OR (95% CI)	*p*	*p*_h_/*I*^2^	OR (95% CI)	*p*	*p*_h_/*I*^2^
**All**	28	17,479/19,799	1.16 (1.07–1.25)	**0.000**	0.000/79.335	1.28 (1.10–1.50)	**0.002**	0.000/74.804	1.21 (1.09–1.34)	**0.000**	0.000/75.122	1.24 (1.11–1.39)	**0.000**	0.000/80.158	1.17 (1.05–1.30)	**0.005**	0.000/60.832
**Ethnicity**																	
**Caucasian**	8	2135/4184	1.20 (0.99–1.45)	0.053	0.000/79.874	1.45 (1.02–2.08)	**0.040**	0.000/78.000	1.03 (0.75–1.40)	0.877	0.000/78.481	1.16 (0.84–1.60)	0.373	0.000/82.426	1.46 (1.28–1.66)	**0.000**	0.120/38.884
**Asian**	20	15,344/15,615	1.14 (1.05–1.24)	**0.002**	0.000/79.288	1.22 (1.03–1.45)	**0.020**	0.000/73.249	1.27 (1.14–1.41)	**0.000**	0.000/74.134	1.27 (1.13–1.43)	**0.000**	0.000/80.253	1.08 (0.96–1.22)	0.262	0.001/57.234
**Cancer type**																	
**BC**	4	2386/4250	1.21 (1.12–1.32)	**0.000**	0.992/0.000	1.39 (1.14–1.69)	**0.001**	0.873/0.000	1.37 (1.21–1.54)	**0.000**	0.576/0.000	1.36 (1.22–1.53)	**0.000**	0.689/0.000	1.12 (0.945–1.33)	0.192	0.848 /0.000
**GC**	16	11,483/10,882	1.21 (1.09–1.35)	**0.000**	0.000/83.251	1.45 (1.16–1.78)	**0.001**	0.000/80.616	1.30 (1.16–1.45)	**0.000**	0.000/62.932	1.36 (1.19–1.55)	**0.000**	0.000/76.780	1.25 (1.07–1.47)	**0.007**	0.000/72.933
**Other Cancer**	8	3610/4667	0.96 (0.90–1.03)	0.291	0.218/26.402	0.95 (0.81–1.11)	0.481	0.321/13.908	0.90 (0.72–1.11)	0.312	0.013/60.713	0.92 (0.75–1.11)	0.383	0.019/58.256	1.02 (0.89–1.17)	0.765	0.552/0.000

Significant associations are shown in bold, ***p***h—*p* value of Q test for heterogeneity, OR—Odds Ratio, CI—Confidence Interval.

**Table 4:** Meta-analysis Result for *PSCA* rs2976392 *G* > *A* polymorphism.

Variables	*N*	Case/Control	*A vs.* *G* Allele			*AA vs.* *GG*			*GA vs.* *GG*			*GA* + *AA vs.* *GG*			*AA vs.* *GG* + *GA*		
			OR (95% CI)	*p*	*p*_h_*/I*^2^	OR (95% CI)	*p*	*p*_h_/*I*^2^	OR (95% CI)	*p*	*p*_h_/*I*^2^	OR (95% CI)	*p*	*p*_h_/*I*^2^	OR (95% CI)	*p*	*p*_h_/*I*^2^
**All**	11	5970/5707	1.09 (0.95–1.25)	0.210	0.000/81.116	1.10 (0.82–1.48)	0.654	0.000/78.184	1.13 (0.98–1.31)	0.087	0.004/61.549	1.161 (0.93–1.33)	0.256	0.000/77.837	1.04 (0.82–1.32)	0.756	0.000/73.621
**Ethnicity**																	
**Caucasian**	2	431/614	1.39 (0.77–2.51)	0.281	0.001/90.899	1.90 (0.60–6.03)	0.276	0.001/90.399	1.20 (0.57–2.54)	0.639	0.020/81.566	1.22 (0.36–4.19)	0.753	0.000/94.07	1.66 (0.88–3.13)	0.120	0.024/80.307
**Asian**	9	5053/4612	1.04 (0.91–1.19)	0.540	0.000/76.841	0.981 (0.75–1.28)	0.891	0.001/69.334	1.13 (0.98–1.31)	0.100	0.008/61.119	1.10 (0.94–1.30)	0.242	0.000/71.634	0.93 (0.75–1.16)	0.529	0.010/60.305
**Cancer type**																	
**GC**	7	5087/4524	1.14 (0.95–1.38)	0.165	0.000/87.766	1.16 (0.76–1.77)	0.498	0.000/86.220	1.21 (1.02–1.43)	**0.026**	0.003/69.157	1.22 (0.99–1.50)	0.066	0.000/82.190	1.04 (0.73–1.48)	0.829	0.000/82.477
**Other Cancer**	4	873/1183	1.02 (0.90–1.16)	0.264	0.428/0.000	1.05 (0.82–1.35)	0.713	0.000/0.520	0.91 (0.74–1.14)	0.419	0.676/0.000	0.87 (0.71–1.07)	0.189	0.210/33.715	1.10 (0.89–1.36)	0.365	0.349/8.804

Significant associations are shown in bold, ***p***h—*p* value of Q test for heterogeneity, OR—Odds Ratio, CI—Confidence Interval.

For rs2976392 polymorphism, the removal of the studies by Matsuo *et al.* (2009) [27], Kupcinskas *et al.* (2014) [53] collectively abolished heterogeneity in all log additive and recessive genonotypic model (*AA vs.* *GG*: *p*h = 0.928, *I*^2^ = 0.000; *GA vs.* *GG*: *p*h = 0.150, *I*^2^ = 33.533; *AA vs.* *GG* + *GA*: *p*h = 0.893, *I*^2^ = 0.000; *A vs.* *G*: *p*h = 0.586, *I*^2^ = 0.000). However, dominant model required the removal of Matsuo *et al.* (2009) [27], Kupcinskas *et al.* (2014) [53] and Kupcinskas *et al.* (2015) [58] to remove heterogeneity (*GA* + *AA vs.* *GG*: *p*h = 0.328, *I*^2^ = 13.123).

### 3.4. Publication Bias

*DCC* rs714 polymorphism showed funnel plot symmetry in all genetic models. Egger’s test (*AA vs.* *GG*: *t* = 0.146, *p* = 0.893, Figure 2B.; *GA vs.* *GG*: *t* = 0.275, *p* = 0.801; *GA* + *AA vs.* *GG*: *t* = 0.569, *p* = 0.609; *AA vs.* *GA* + *GG*: *t* = 0.153, *p* = 0.566; and *A vs.* *G* allele: *t* = 0.875, *p* = 0.446) as well as Begg and Mazumdar rank correlation (*AA vs.* *GG*: *p*_2tailed_ = 893; *GA vs.* *GG*: *p*_2tailed_ = 0.807; *GA* + *AA vs.* *GG*: *p*_2tailed_ = 0.807; *AA vs.* *GA* + *GG*: *p*_2tailed_ = 1.000 and *A vs.* *G* allele: *p*_2tailed_ = 0.221) also confirmed the funnel plot symmetry.

**Figure 5:**
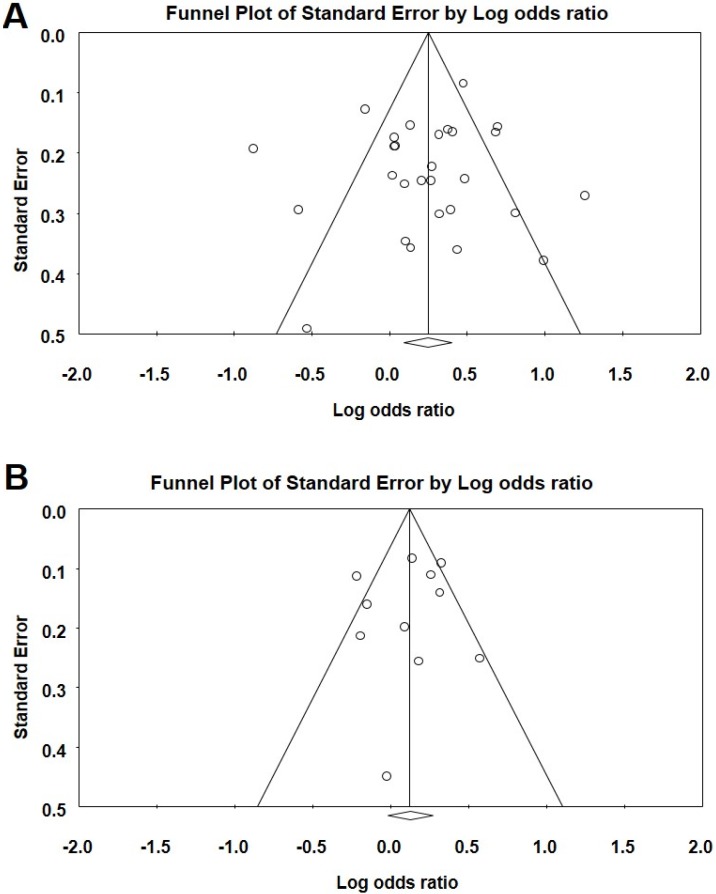
(**A**) Funnel plot analysis to detect publication bias for the *PSCA* rs2294008 *C* > *T* polymorphism (TT *vs.* CC). Each dot represents an individual study for the indicated association. Areas of squares of individual studies are inversely proportional to the variance of the log odds ratios and the horizontal lines represent CIs; (**B**) Funnel plot analysis to detect publication bias for the *PSCA* rs2976392 *G* > *A* polymorphism (*GA* *vs.* GG). Each dot represents an individual study for the indicated association. Areas of squares of individual studies are inversely proportional to the variance of the log odds ratios and the horizontal lines represent CIs.

For *PSCA* polymorphism, a review of funnel plot also demonstrated no apparent asymmetry in all comparison models; in the overall and subgroup meta-analysis (Figure 5). Egger’s test also did not indicate any evidence of publication bias and statistically establish the funnel plot symmetry (for rs2294008—*TT vs.* *CC*: *t* = 0.466, *p* = 0.645; *CT vs.* *CC*: *t* = 0.573, *p* = 0.572; *CT* + *TT vs.* *CC*: *t* = 874, *p* = 0.390; *TT vs.* *CT* + *CC*: *t* = 0.634, *p* = 0.549; and *T vs.* *A* allele: *t* = 0.351, *p* = 0.728, and for rs2976392 SNP, *AA vs.* *GG*: *t* = 0.349, *p* = 0.735; *GA vs.* *GG*: *t* = 0.387, *p* = 0.708; *GA* + *AA vs.* *GG*: *t* = 0.261, *p* = 0.800; *AA vs.* *GA* + *GG*: *t* = 0.133, *p* = 0.897; and *A vs.* *G* allele: *t* = 0.150, *p* = 0.884). Similarly, Begg and Mazumdar rank correlation test also did not indicate any publication bias (for rs2294008—*TT vs.* *CC*: *p*_2tailed_ = 0.921; *CT vs.* *CC*: *p*_2tailed_ = 0.678; *CT* + *TT vs.* *CC*: *p*_2tailed_ = 0.890; *TT vs.* *CT* + *CC*: *p*_2tailed_ = 0.387 and *T vs.* *A* allele: *p*_2tailed_ = 0.621 and for rs2976392 *AA vs.* *GG*: *p*_2tailed_ = 0.876; *GA vs.* *GG*: *p*_2tailed_ = 0.756; *GA* + *AA vs.* *GG*: *p*_2tailed_ = 0.756; *AA vs.* *GA* + *GG*: *p*_2tailed_ = 1.000 and *A vs.* *G* allele: *p*_2tailed_ = 0.756) suggesting that our results were statistically robust.

## 4. Discussion

In the present meta-analysis, we found that DCC rs714 conferred a significantly increased risk of cancer only in Asians. Previously, it was shown to be associated with GBC, GC and EC in Asian population [10,12]. Toma *et al.* (2009) showed increased risk of CRC in Caucasians [34], though our meta-analysis is limited for Caucasian ethnicity due to lack of published data. On the other hand, a study by Djansugurova *et al.* (2015) involving mixed population showed that A allele of rs714 confers protection against the CRC risk [13].

We also established that the PSCA rs2294008 polymorphism is significantly associated with increased cancer susceptibility, overall. However, the estimate of the association is predominantly determined by that for gastric and bladder cancer as we failed to detect the association of *PSCA* rs2294008 polymorphism with other cancer risk. This may be ascribable to the fact that different cancer has a different molecular mechanism of the disease process and the number of studies is limited in other cancer subgroups. Further, the significant association of this polymorphism with cancer was more prominent in Asians as compared to Caucasians. A previous meta-analysis also showed that *PSCA* rs2294008*C* > *T* polymorphism is closely associated with increased risk of GC for Eastern Asians [59]. Though we have excluded GWAS studies from our meta-analysis, our results are in agreement with the previous GWAS studies [22,23,60] and meta-analysis investigating the association of rs2294008 polymorphism with cancer risk, including gastric [59,61,62,63,64,65,66] and bladder cancer [67,68]. These findings suggested rs2294008 as a most promising genetic marker for GC and BC susceptibility. Although, the exact mechanism of PSCA to promote carcinogenesis remains unclear, its expression has been associated with the malignant progression of pre-malignant lesions and advanced clinical stage and metastasis in prostate cancer [25]. The rs2294008 *C* > *T* is located in exon 1, and *in* vitro experiments have revealed that the variant is associated with the reduced transcriptional activity of an upstream fragment of *PSCA* [22,35].

In contrast, we did not find any association of *PSCA* rs2976392 SNP with cancer risk. Previously, heterogenotype and dominant model of rs2976392 was found to confer significantly increased risk of GC, specifically in females or non-cardia GC [36,54]. Lu *et al.* (2010) also showed a significant association of this SNP with GC risk [37] while other studies showed no association [41,44,47]. Moreover, previous meta-analysis also demonstrated that the rs2976392 SNP conferred increased risk of GC [59,61,62,63,65,68]. This discrepancy may be due to the inclusion of GWAS studies which is the largest number of association studies dominating the result of pooled analysis in all previous meta-analysis. The rs2976392*G* > *A* positioned in intron 2 is in strong linkage disequilibrium with rs2294008*C* > *T*, and its function is unclear till yet [61]. Hence, the positive association observed in various case-control studies may be due to the linkage effect of rs2294008 polymorphism.

Our study is the most up-to-date study, including all the published case-control studies in English language until September 2015. However, like other studies, we also have some flaws such as; possibility of selection biases due to study selection based on English language only and different genotyping methods. Likewise, the number of available studies were not sufficient in subgroup analysis, such as for other cancers, Cacuasians and mixed populations to perform a comprehensive analysis. Thus, our study is not a complete cancer analysis but is limited to the Asian population and specific cancer (BC and GC) for rs714 and rs2294008 polymorphism, respectively. Furthermore, our association analysis was based on unadjusted or crude estimates and the roles of gene-gene, gene-environment interactions, as well as linkage disequilibrium was not considered. Further analysis considering all these is required to make a more appropriate association of *DCC* and *PSCA* gene variants in modulation of cancer risk.

### Study Advantage

Since we pooled large number of cases and controls from various studies, our study has improved statistical power of the analysis. In addition, we adopted a stringent inclusion/exclusion touchstone to include the well-defined case-control studies in the present meta-analysis. We have excluded GWAS thus preventing the likely bias. We also performed sensitivity analysis confirming the stability of our meta-analysis results in all models.

## 5. Conclusions

Our meta-analysis results showed that *DCC* rs714 polymorphism was associated with increased risk of cancer in Asian populations. Further, we confirmed a firm association between the *PSCA* rs2294008 *C* > *T* polymorphism with increased susceptibility of gastric and bladder cancers, signifying PSCA rs2294008 polymorphism as potential biomarker for these cancers. However, since our study is limited for ethnicity (*DCC* rs714) and cancer types (*PSCA* rs2294008), further larger studies involving other cancers and other population are needed to perform a more rigorous comparative analysis to corroborate this conclusion and to assess the more accurate association between these polymorphisms and overall cancer risk.

## Abbreviation

PSCA	Prostate stem cell antigen
DCC	Deleted in Colorectal Carcinoma
SNP	Single nucleotide polymorphism
GBC	Gallbladder cancer
GC	Gastric cancer
EC	Esophageal cancer
BC	Bladder cancer
PC	Prostate cancer
CRC	Colorectal cancer
BrC	Breast cancer
OR	Odds ration
CI	Class interval
HWE	Hardy Weinberg Equilibrium
GWAS	Genome wide association study
PCR-RFLP	Polymerase chain reaction-restriction fragment length polymorphism
LDR	Ligation detection reaction
HRM	High-resolution melting (HRM)
